# Mr. Sander’s Mission

**Published:** 1883-07

**Authors:** 


Mr. J. H. Sanders, of the *Breeder's Gazette*, member of the United States Cattle Commission, has left for Europe on a special mission by appointment from the department of Agriculture at Washington, in reference to the International Live Stock Show at Hamburg, to our cattle trade with Great Britain and other European countries, to the crusade in Germany and elsewhere on the continent against American pork, and to a general review of the live stock interests of Europe as compared with our own. He will visit the principal live stock markets and breeding districts of Europe, and may give the readers of the *Gazette* the result of his observations in occasional letters while abroad.

